# Eating for Two: Does an Organic Diet Make a Difference?

**DOI:** 10.1289/ehp.124-55

**Published:** 2016-03-01

**Authors:** Carol Potera

**Affiliations:** Carol Potera, based in Montana, also writes for *Microbe*, *Genetic Engineering News*, and the *American Journal of Nursing*.

The organic food market is one of the fastest growing sectors of the U.S. and European agricultural industries,^1^^,^^2^ but few conclusions have been reached regarding whether organic foods are actually better for consumers than their conventional counterparts. A study in this issue of *EHP* probed that question and estimated that women who ate organic food during pregnancy were 58% less likely to deliver boys with hypospadias, a common urogenital birth defect, than mothers who never ate any.^3^

The study analyzed 35,107 women and their male infants who participated in the Norwegian Mother and Child Cohort Study. The women filled out a food frequency questionnaire that asked about their consumption of six categories of organic food (vegetables, fruits, cereals, milk/dairy, eggs, and meat) during the first four months of pregnancy.

**Figure:**
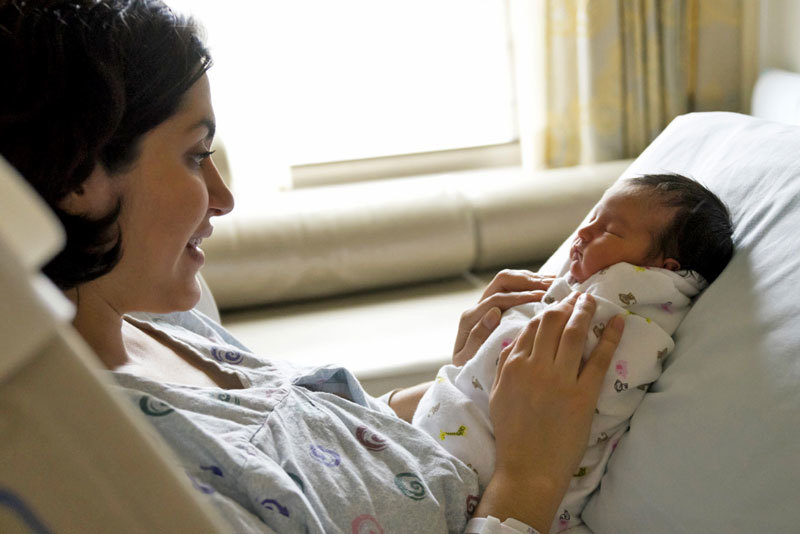
Women who ate organic vegetables during pregnancy had fewer babies with hypospadias, but it’s unclear to what degree, if any, the choice of organic food was responsible. Organic or not, it’s important for pregnant women to eat plenty of produce. © Ariel Skelley/Getty

About 48% of the women reported eating at least one of the organic food groups “often/mostly” or “sometimes.” The women in these two groups gave birth to 22 infants with hypospadias. The other half of the women reported “never/seldom” eating organic foods, and 52 cases of hypospadias occurred in these mothers. Of all the food groups, organic vegetables had the strongest association with lower prevalence of hypospadias.^3^

The researchers also assessed prevalence of cryptorchidism, another common birth defect in boys. They found no association between cryptorchidism among the boys and organic food consumption by their mothers.^3^

In hypospadias, the urinary opening of the urethra forms on the underside, rather than at the head, of the penis. If not corrected surgically, hypospadias may impair fertility or lead to testicular cancer.^4^ The prevalence of hypospadias appears to have been rising in some areas, by one estimate affecting 4–6 boys per 1,000 male births.^5^

Hypospadias develops during weeks 8–14 of pregnancy, and its causes are unknown.^4^ The development of external male genitalia is a complex process that involves pathways susceptible to genetic, endocrine, and environmental factors. Because many pesticides contain endocrine-active chemicals,^6^ it has been suggested that reduced pesticide exposures among pregnant women who eat organic food may protect their unborn children against endocrine-related health effects such as hypospadias. However, this remains a “purely hypothetical suggestion,” says study leader Anne Lise Brantsæter, a senior scientist at the Norwegian Institute of Public Health in Oslo.

Preeclampsia also is associated with increased risk for hypospadias.^7^ Previously, Brantsæter and her colleagues showed reduced incidence of preeclampsia among pregnant women who ate organic vegetables, compared with those who didn’t.^8^

Strengths of the study include its prospective design and large cohort. It is limited by the lack of detailed information on diet and family history of hypospadias, as well as lack of biospecimen data, which would indicate whether eating organic produce actually translated into lower pesticide exposures. The authors caution that their findings were based on small numbers of cases and require replication in other study populations. In addition, selecting organic food may be a proxy for other behaviors that would reduce exposures to endocrine-disrupting chemicals, such as choices in cleaning products and personal care products.^3^

According to Cynthia Curl, an assistant professor in the Department of Community and Environmental Health at Boise State University, there’s little evidence to support or dispute the perception by some consumers that organic food is healthier than conventional alternatives. “We need epidemiological research like Brantsæter’s to provide consumers with information they can use to decide when and what organic food to buy,” says Curl, who was not involved in the study.

In the meantime, Brantsæter recommends that all pregnant women eat a balanced diet that includes at least five servings of fruits and vegetables daily, regardless of whether the produce is organic or not. “The beneficial health effects of vegetables and fruits are well established,” she says, “whereas the jury is still out on the benefits of organic alternatives.”
